# Preparation and Characterization of Biocomposite Films with Enhanced Oxygen Barrier and Antioxidant Properties Based on Polylactide and Extracts from Coffee Silverskin

**DOI:** 10.3390/molecules30061383

**Published:** 2025-03-20

**Authors:** Argyri-Ioanna Petaloti, Adamantini Paraskevopoulou, Dimitris S. Achilias

**Affiliations:** 1 Laboratory of Polymer and Color Chemistry and Technology, Department of Chemistry, Aristotle University of Thessaloniki, 54124 Thessaloniki, Greece; apetaloti@chem.auth.gr; 2 Laboratory of Food Chemistry and Technology, Department of Chemistry, Aristotle University of Thessaloniki, 54124 Thessaloniki, Greece; adparask@chem.auth.gr

**Keywords:** coffee silverskin, PLA, biocomposites, circular economy, antioxidant activity, food packaging

## Abstract

In the food packaging industry, significant efforts have been dedicated to addressing the pressing market demand for environmentally friendly and sustainable products. Biocomposite films based on compostable and biobased polymers represent a sustainable alternative to conventional packaging materials, offering biodegradability and enhanced functional properties. Additionally, there is growing interest in utilizing waste materials from agriculture and the food industry. This study focuses on the development of multifunctional eco-sustainable biocomposite films by combining poly(lactic acid) (PLA) as a biopolymeric matrix and extracts derived from coffee silverskin (CS), a significant agro-industrial waste byproduct of the coffee roasting process. Extracts of coffee silverskin were obtained via extraction with several solvents. Several properties of the prepared biocomposites were measured using techniques such as scanning electron microscopy (SEM), infrared spectroscopy (FTIR, ATR), differential scanning calorimetry (DSC), and oxygen and water vapor permeability, together with mechanical and physico-chemical characterization and measurements of water content, film solubility, and degree of swelling. The results demonstrate that optimized formulations of PLA/CS biocomposite films exhibit enhanced oxygen barrier properties, reduced permeability, and significant antioxidant activity. These findings underscore the potential for agro-waste valorization in creating eco-friendly food packaging solutions.

## 1. Introduction

The utilization of plastics has been on a steady rise over the years owing to their desirable properties, with global production reaching 338 million tons in 2019, i.e., a rise of up to 640% since 1975 [1]. Despite the potential benefits of enhancing resource circularity and mitigating environmental impacts associated with production, the recycling rate for plastics remains inadequate [2]. The majority of plastics exhibit a lack of biodegradability, and their complete decomposition can take over a hundred years [3]. Between 1950 and 2015, it is estimated that 80% of plastic waste was disposed of in landfills or natural environments [4].

There exist three distinct approaches to mitigating the quantity of polymer waste present in the environment. One potential solution to address the issue of plastic waste is to decrease the production of polymer materials, but this approach is challenging to envision, as the production of such materials continues to rise annually, and this trend will be difficult to reverse in the foreseeable future. A second approach is to enhance the plastic waste recycling process, a strategy that is gaining traction and visibility worldwide, not solely in highly developed nations. A third solution is to promote the adoption of biodegradable polymers, particularly those derived from renewable sources. Combining the latter two methods could effectively reduce residual waste and yield positive environmental outcomes [5].

The primary benefit of biodegradable polymers lies in their ability to undergo complete degradation within a relatively short period of time, typically a few months, when exposed to suitable environmental conditions [6]. The use of bio-based polymers offers several environmental advantages, including reduced greenhouse gas emissions, lower quantities of hazardous chemicals and pollutants, and the conservation of ecosystems and biodiversity, among others [7,8]. Representative bio-based polymers include poly(lactic acid) (PLA), poly(butylene succinate) (PBS), poly(ethylene furanoate) (PEF), and polyhydroxyalkanoates (PHAs). The worldwide production of these polymers is expected to increase from approximately 2.11 million tons in 2019 to about 2.43 million tons by 2024 [9]. PLA, a bio-based polymer derived from renewable resources, has gained prominence in this domain due to its excellent mechanical properties and compostability. However, its inherent limitations, such as high oxygen permeability and low functional activity, restrict its application in certain sectors, particularly food packaging.

The escalation of solid waste generation, encompassing agricultural, industrial, household, human, and animal waste, has emerged as a significant issue that leads to environmental contamination on a global scale. In response to these challenges, the utilization of biotechnological methodologies for waste valorization is gaining momentum as a sustainable and eco-friendly solution to address this issue [10]. Coffee, one of the most widely consumed beverages globally, is ranked as the second-largest traded commodity following petroleum, underscoring its significant market presence [11]. The coffee industry produces over 10 million tons of waste worldwide annually [12]. This waste encompasses a range of byproducts, including husks, pulp, mucilage, silverskin, and spent coffee grounds (SCGs), which are generated during various stages of coffee processing, including harvesting, processing, roasting, and brewing [13].

The food packaging industry is currently witnessing a growing need for the reduction or complete elimination of synthetic additives. To address microbial growth and food oxidation, the incorporation of natural food additives, such as essential oils or plant extracts, presents a viable solution [14]. Coffee silverskin (CS), a byproduct of the coffee bean roasting process, constitutes a mere 4.3% (*w*/*w*) of the coffee cherry and, despite its relatively small proportion, it is noteworthy for its high dietary fiber content (80%) and its abundance of antioxidants and phenolic compounds [11,15,16]. The beneficial functional and nutritional properties of these phenolic compounds have garnered significant attention, particularly for their antioxidant and antimicrobial activities [14]. Furthermore, recent research has demonstrated that CS contains a diverse range of bioactive compounds, including melanoidins, caffeine, and polyphenols. These compounds show promising potential for use as functional ingredients in cosmetic and nutraceutical formulations [17,18]. Therefore, CS can be used as a source for extracting antioxidants, owing to its elevated chlorogenic acid levels [19,20]. The lipid fraction of CS, ranging from 2.4% to 3.4%, mainly consists of linoleic, palmitic, behenic, and arachidic acids, while also holding potential as a source of bioactive compounds like phytosterols [21,22]. By incorporating CS extracts into PLA, it is possible to enhance the functional properties of the resultant films, particularly oxygen barrier and antioxidant capacities, thereby extending their shelf-life potential and reducing food spoilage.

In this study, the production process for a novel active food packaging solution was optimized. Specifically, various concentrations of CS extracts were incorporated into a PLA-based matrix, presenting a sustainable alternative to traditional packaging by utilizing a biodegradable polymer and promoting waste valorization. Active films were fabricated using solvent casting and characterized in terms of morphology, color, and antioxidant activity. Following this, a physico-chemical characterization of the films was conducted, which included assessment of their water content, solubility, swelling degree, color measurements, and oxygen and water transition rate. The films were further characterized according to their structural, mechanical, and functional properties.

## 2. Results and Discussion

### 2.1. Morphological and Structural Observations

After extraction of coffee silverskin oil using a Soxhlet apparatus with hexane solvent, the freshly extracted oil was dark brown in color, having a mild coffee odor. In this study, the yield of oil extracted with 100% hexane (nonpolar solvent) was 3.6 ± 0.3% in 5 h of extraction time from Soxhlet extraction, similar to that reported by other researchers (i.e., 3.8 ± 0.4% [23], 2.4% [22], and 3.4% [21]). The disparity observed in the lipid composition of coffee silverskin can be ascribed partially to various factors, including the distinct combinations of coffee types (Arabica and Robusta), the source of the coffee beans (cultivation climate, harvesting), and the technique employed for processing (wet or dry processing and roasting) [24]. Lipids are soluble in organic solvents but have limited solubility in water. Using polar solvents for extraction leads to lower oil yields compared to non-polar solvents [25,26].

**Figure 1:**
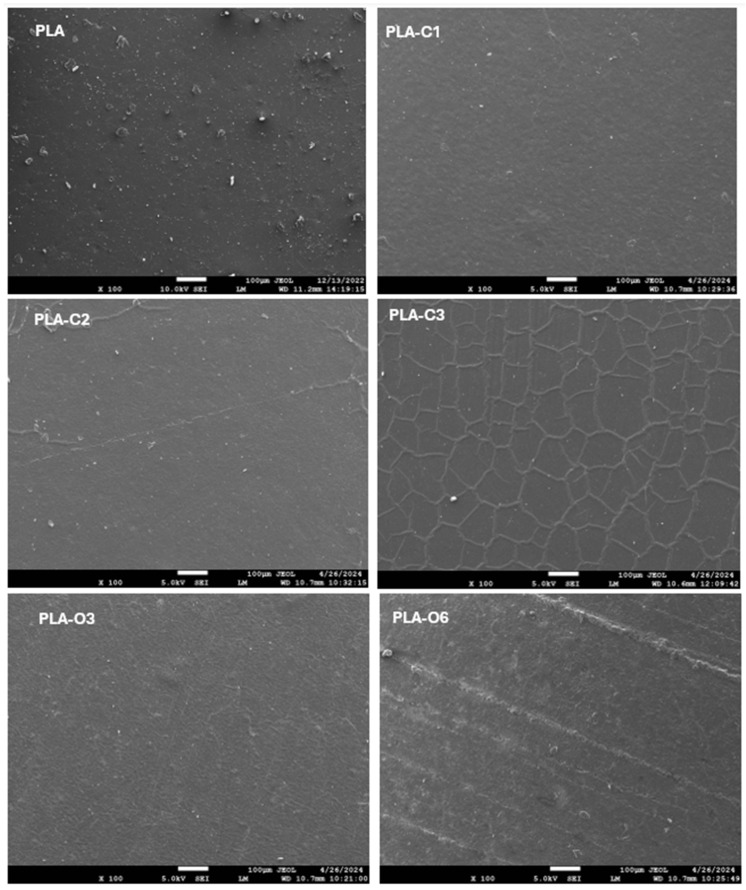
SEM photographs of PLA films with chloroform extract and oil extract of coffee silverskin. The photos illustrate the surface morphology and texture of the films.

Figure 1 presents SEM photos of pure PLA and PLA films containing coffee silverskin extracts. The films with lower chloroform extract concentrations (PLA-C1 and PLA-C2), exhibited a more continuous and robust structure, revealing a homogeneous dispersion of CS extracts in the polymer matrix. Conversely, film PLA-C3 displayed a scaly and flake-like structure, while no visible cracks were present, indicating that the integration of CS chloroform extract at higher concentrations was not fully successful. Notably, films with coffee silverskin oil extracts demonstrated a more uniform and seamless structure, without any breaks or interruptions. This implies a possible interaction between the PLA chains and the CS extracts, particularly with the fatty acids present in the coffee silverskin extracts. Similar results were observed in a previous investigation, where films based on PLA and spent coffee grounds extract exhibited a distinctive flake-like, cauliflower structure, attributed to interactions between the fatty acid present within coffee extract and the PLA chains [5].

**Figure 2:**
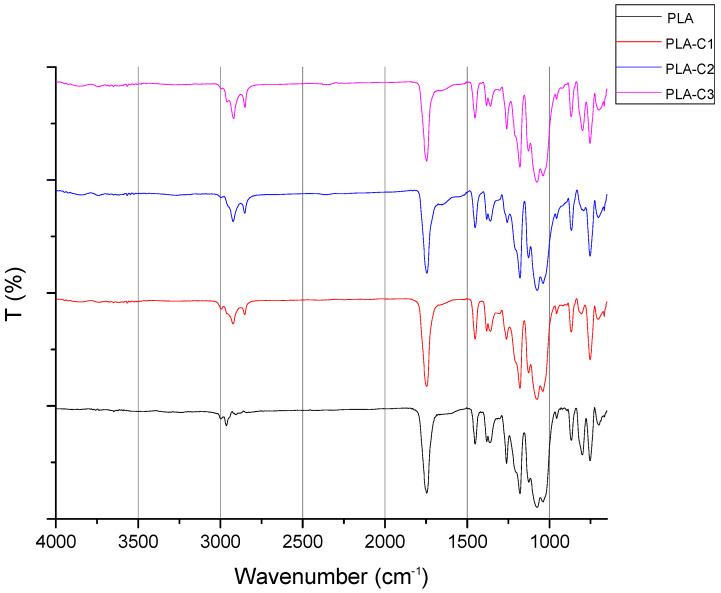
FTIR/ATR spectra of PLA and composites with CS chloroform extracts.

Figure 2 shows the FTIR spectrum of PLA and films containing CS chloroform extracts (i.e., PLA-C1, PLA-C2, and PLA-C3). The IR spectrum of PLA indicates a prominent peak at 1750 cm^−1^, which corresponds to the stretching of the C=O bond, while the peaks at 2800 cm^−1^ and 2950 cm^−1^ are attributed to the stretching of C-H bonds in the -CH_3_ group. A distinctive absorption peak for ester C-O stretching occurs at 1200 cm^−1^. The FTIR spectrum of PLA aligns with the IR spectra documented in previous literature [27,28]. Additional peaks are observed in the films with CS extract, alongside the typical PLA vibrational modes, i.e., a peak around 1650 cm^−1^, which can be related to the presence of chlorogenic acids and caffeine, or peaks at 2920 cm^−1^ and 2850 cm^−1^ that further confirm the presence of CS extract in the polymer extract. In particular, the peak at 1650 cm^−1^ is attributed to the vibration of the carbonyl (C=O) group in triglycerides [29] or aliphatic esters [30]. Additionally, the two distinct peaks at 2920 cm^−1^ and 2850 cm^−1^ suggest the presence of caffeine [31,32] and lipids [33]. Also, it can be observed that the increase in extract content led to stronger intensities in the regions 2850, 2900, and 1650 cm⁻^1^. Notably, these peaks were also observed in the spectra of PLA films containing spent coffee ground extracts [34].

**Figure 3:**
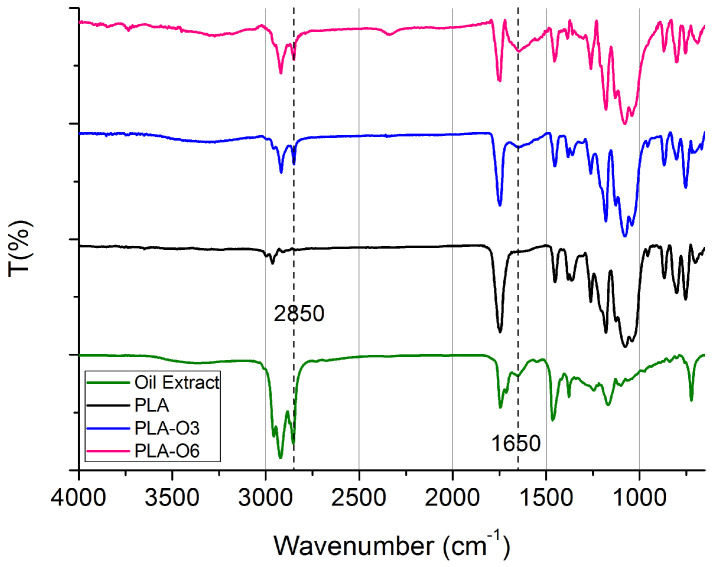
FTIR/ATR spectra of PLA composites with CS oil extracts.

The FTIR spectrum in Figure 3 shows the main functional groups identified in the films containing CS oil, i.e., PLA-O3 and PLA-O6. In the CS oil, the broad peak around 3400 cm^−1^ corresponds to the O-H stretching vibrations from the hydroxyl groups, such as those found in free glycerol and water. The strong bands at 2850 cm^−1^ and 2900 cm^−1^ are linked to the asymmetric and symmetric stretching vibrations of CH_2_, which are characteristic of the fatty acids present in the oil [35]. The region between 1750 cm^−1^ and 1710 cm^−1^ reveals the ester carbonyl stretching vibration of triglycerides (O-C=O), as well as the carbonyl (C=O) stretching vibration of free fatty acids [36]. The stretching vibration of the carbonyl group (C=O), which is also connected to caffeine and chlorogenic acids, is responsible for the peak at about 1650 cm^−1^. The stretching and rocking vibration of the C–O ester group is indicated by the peak at 1200 cm^−1^, while the bending vibration of the C–H of the CH_2_ and CH_3_ aliphatic groups is represented by the peak at 1480 cm^−1^. The 720 cm^−1^ zone is where the out-of-plane vibration of cis-disubstituted olefins and the aliphatic CH_2_ rocking vibration overlap. Furthermore, the increased addition of the coffee silverskin oil extract led to more intense absorption bands at 3400, 2850, 2900, 1650, and 1200 cm⁻^1^. In general, the FTIR spectra of coffee oil show characteristic signals across the entire region, consistent with previous studies [37]. When comparing the FTIR spectra of PLA before and after the addition of CS oil, the most notable difference was the increased peak intensity in the same regions corresponding to significant peaks in the CS oil (i.e., 3400 cm^−1^, 1650 cm^−1^). This enhancement confirms the successful integration of CS oil into the PLA matrix. Similar results were achieved when spent coffee ground oil was incorporated into a PLA polymer matrix [38].

### 2.2. Color Measurements

**Figure 4:**
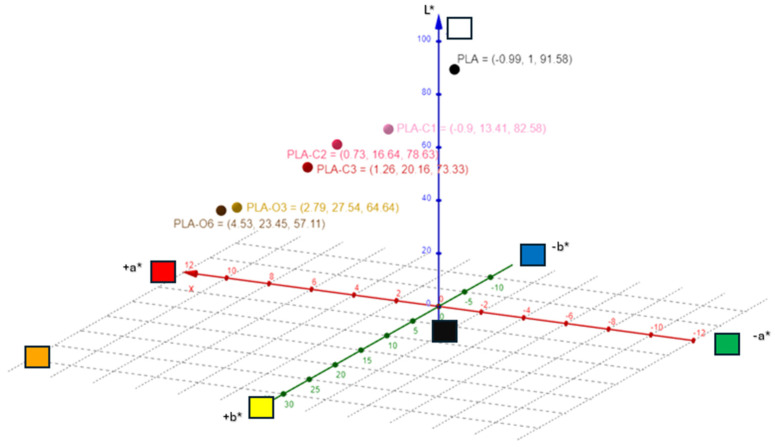
Color characteristics of the composite materials studied.

As shown in Figure 4 and Table 1, the color of the PLA films changed after the addition of CS extracts. The lightness (L*) values were lower for the PLA composites compared to pure PLA film. A gradual decrease in lightness was observed across all samples, with a 19.93% decrease for the film containing the highest concentration of CS chloroform extract (PLA-C3) and a 37.64% decrease for the PLA-O6 film with the highest addition of CS oil. As for the a* values, they gradually increased, and higher values were recorded for the films containing CS oil extract. On the contrary, the a* values of films with CS chloroform extracts did not increase as significantly, as expected. In the case of the b* and c* values, all films containing extracts showed a progressive increase in both b* and c* values. H* values were also decreased upon CS extract addition, up to 36.66% for PLA-C3 and 42.05% for PLA-O6 compared to the pure PLA film. Increasing the concentration of CS extracts led to either a yellowish or brownish-yellow appearance in films with CS chloroform or oil extracts, respectively. The gradual incorporation of the extracts resulted in increased color strength values, with notably higher levels for the CS oil-containing films. Coffee silverskin as filler in PLA matrix gave a brownish appearance to films [39]. Coffee and cocoa extracts gave PLA films a brown coloration, with intensity varying by extract type and concentration; coffee extract resulted in the lightest color, while cocoa extract produced the darkest hue [5]

### 2.3. Thermal Behavior

**Figure 5:**
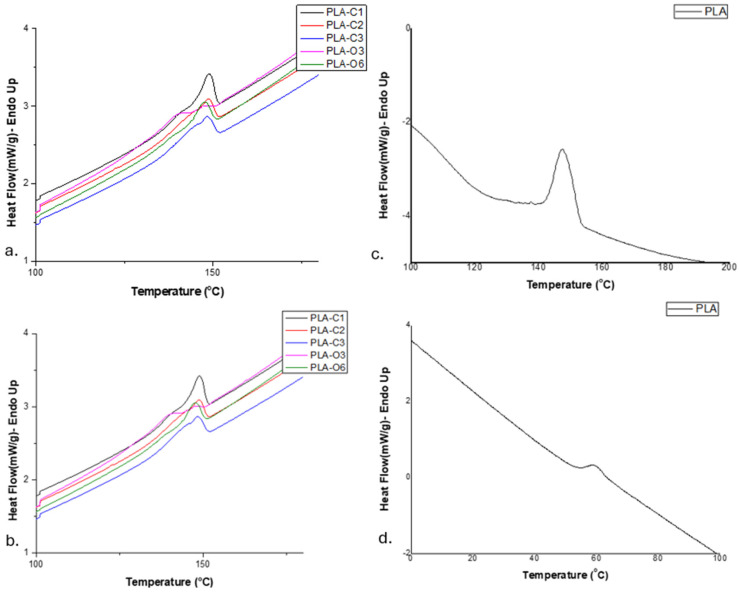
DSC scans of the biocomposite films studied: (**a**) first heating of films with CS extracts; (**b**) second heating of films with CS extracts; (**c**) first heating of pure PLA film; (**d**) second heating of pure PLA film.

Differential scanning calorimetry was employed to assess the thermal behavior of the films. Two subsequent heating scans were applied to each sample. The glass transition temperature (T_g_) of the polymer in the composites could not be clearly determined from the first heating scan, so results from the second heating scan were used. On the other hand, the melting point (T_m_) was determined from the first heating scan, as it was not detectable in the second one. It is also noteworthy in Figure 5 that the cold-crystallization temperature (Tcc) of PLA in the films was not observable (i.e., no exothermic peak appeared). The heat of fusion measured was around 17 to 18 J/g for the PLA-C series, whereas it was much lower, i.e., 9.5 to 13, for the PLA-O composites (Table 2).

T_g_ is known to be influenced by various factors, including intermolecular interactions, chain flexibility, and the molecular weight of the material [40]. According to the data reported in Table 2, the addition of CS extracts did not affect the Tg value of the polymer, which remained at about 57–58 °C for all the films (Figure 5, Table 2), similarly to the value 56.9 estimated for neat PLA [39].

The extract’s components, incorporated during the solvent casting process, favored the rearrangement of PLA chains, resulting in a more organized crystalline structure. These components acted as “crystallization nuclei”, as evidenced by their influence on the overall crystallization behavior of the PLA chains [34]. Furthermore, only a single melting peak with a broad shoulder was observed for almost all composites. The melting peak ranged from 146.7 to 148.6 °C, similar to that measured for neat PLA, i.e., 146.8 °C [39]. In the case of PLA-O3, the shoulder revealed a second distinct melting peak at 139.8 °C together with that at 146 °C (Figure 5). The presence of these two melting peaks can be regarded as evidence for the simultaneous existence of two distinct crystalline structures in PLA, which aligns with the melt recrystallization model [41,42]. Conversely, in PLA-O6, where the oil extract content was doubled, this phenomenon was no longer fully observed, as the peak was not completely uniform—there was a slight tendency for a double peak at the melting temperature. In this case, the higher oil concentration acted as a stronger plasticizer, increasing polymer chain mobility and reducing overall crystallinity, leading to a single melting peak instead. Moreover, the melt recrystallization model appeared to be inhibited due to the higher oil concentration in PLA-O6.

### 2.4. Antioxidant Activity

**Figure 6:**
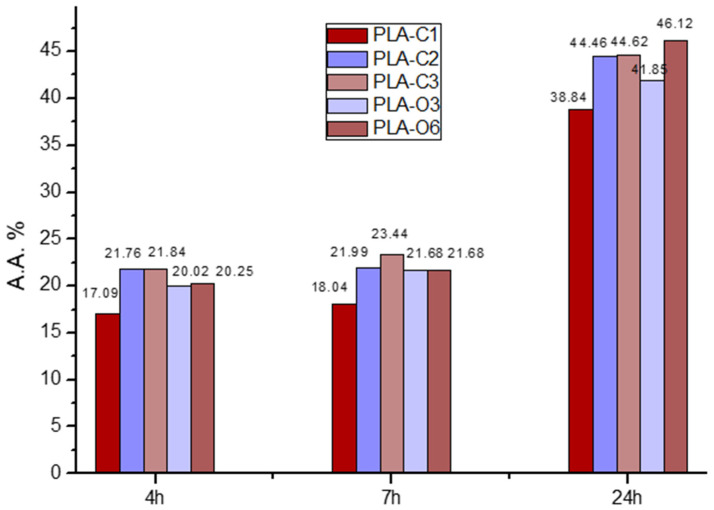
Antioxidant activity of the PLA-based composites with coffee silverskin extracts at time steps 4, 7, and 24 h.

The DPPH method, a popular methodology for evaluating the antioxidant activity of packaging films, was utilized to examine the antioxidant characteristics of the produced films [43]. The samples first showed a purple tint that changed to a yellowish hue, signifying that the addition was having an antioxidant effect. The UV absorbance of the samples was used to measure the color change, and Figure 6 displays the antioxidant activity of the materials that were examined.

All materials demonstrated a peak at 516 nm, as expected. Films containing extracts showed increasing antioxidant activity proportional to the extract percentages. The highest activity was observed in PLA-C2 and PLA-C3 films prepared with 2 and 3% CS extract, as well as in films with the maximum concentration of CS oil (PLA-O6), exceeding 20% after 4 h and 44% after 24 h in all three samples. The antioxidant activity of the composites formed with the CS extracts and presented in Figure 6 was much higher than corresponding values measured in composites of PLA with CS particles, either treated or untreated. The antioxidant activity values measured for the composites of PLA with 5% untreated and treated CS after 3 h were respectively 10.4 and 8.1%, whereas after 24 h, they were 20.3 and 18.1% [39]. Thus the use of extracts resulted in composites having much better antioxidant activity. This activity is likely associated with the composition of the CS extracts and oil, as previously reported. GC-MS analysis of the chloroform extract from spent coffee grounds identified several components, including fatty acids, predominantly C18, that are characteristic organic constituents found in coffee beans, coffee brews, and spent grounds [34,44]. Reported fatty acid percentages revealed that the stearic acid accounted for ~53% of the total fatty acids [34]. Additionally, the caffeine content in spent coffee grounds extract has been reported to be around 2–4.7 mg/g [34,45]. The composition of caffeine, chlorogenic acids, and other molecules differs between Arabica and Robusta species. Different beverage production methods also affect the extraction efficiency of primary antioxidant molecules in coffee beans [46]. All these compounds confer antioxidant activity to the films. The lipid fraction of CS is also known to be rich in bioactive molecules, e.g., phytosterols, which contribute antioxidant properties to the films. The main components of coffee residue oils, such as CS oil, are triacylglycerols, mainly composed of linoleic, palmitic, stearic, and oleic acids [47,48]. Furthermore, high levels of antioxidant activity were produced by coffee silverskin particles and aqueous extracts in a wheat flour–glucose matrix [49]. In addition to providing active packaging for beef and almonds, PLA films containing polyphenolic extracts of green tea and rosemary also produced antioxidant activity in the film matrix [50].

### 2.5. Functional Properties

#### 2.5.1. Gas Barrier Properties

Material barrier properties refer to a material’s capacity to restrict the permeability of vapor and gas. Among the most critical properties that a material must possess to be used in food packaging are the water vapor transmission rate (WVTR) and the oxygen transmission rate (OTR). The results of WVTR and OTR tests of the studied composite films are shown in Table 3. The incorporation of both CS extracts induced a WVTR decrease, with the most significant reduction observed at higher extract concentrations. Specifically, the WVTR value decreased by ~40% for PLA-C3 and PLA-O6 compared with the control film. Water vapor permeability involves both the solubility of water in the film and the diffusion of water molecules through it. The observed reduction in water vapor permeability may be attributed to the lower moisture content in the films caused by addition of the extracts.

The OTR values presented in Table 3 show that the oxygen permeability of pure PLA is 16 cm^3^·mm/m^2^·day·0.1 MPa, aligning with Fukushima et al., who reported a value of 18 cm^3^·mm/m^2^·day·atm [51], though lower than values cited in previous works (e.g., 20 cm^3^·mm/m^2^·day·atm [52]). Oxygen permeability is generally influenced by factors such as chain flexibility, phase and physical state, and packing of polymer’s molecules. In this study, a notable decrease in oxygen transmission rate was observed with increasing CS extract concentrations. Films prepared with the chloroform solution extract showed the lowest oxygen permeability, while films containing CS oil were close to the value of pure PLA. The deep understanding of chemical and physical interactions between CS extracts and the polymer matrix could lead to the development of materials with reduced oxygen permeability, which is essential for food packaging applications requiring high oxygen barrier properties. Materials such as polystyrene (PS), polypropylene (PP), and poly(ethylene terephthalate) (PET) are commonly used in food packaging applications due to their good mechanical and thermal properties. The addition of CS extracts could result in composites with reduced oxygen permeability, thus significantly improving their properties for food packaging applications [53,54].

#### 2.5.2. Water Content, Film Solubility, and Swelling Degree

**Figure 7:**
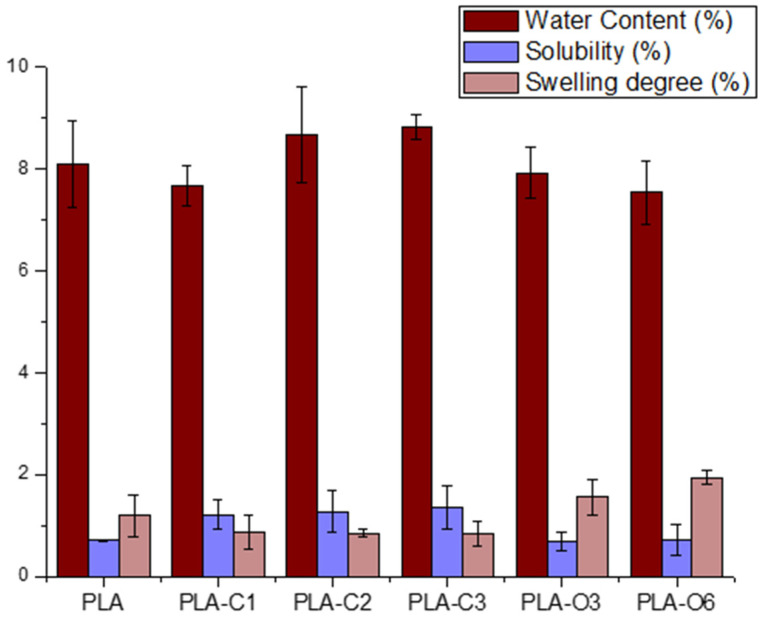
Physical properties of all composites prepared with coffee silverskin extracts.

The basic properties of the films, such as water content, film solubility, and swelling degree, are presented in Table 4 and Figure 7. The water content of films containing CS chloroform extracts was not statistically different compared to neat PLA. The composites with CS oil extracts presented a lower water content compared to PLA, with the smaller value measured for PLA-O6.

The water resistance of biodegradable films is strongly affected by two key factors, i.e., solubility and swelling. These characteristics are very crucial in humid environments, as they determine the film’s capacity to withstand water adsorption and penetration [55]. As shown in Table 4, the PLA control film displayed minimal solubility and limited swelling when immersed in water (25 °C) for 24 h. The addition of CS extract to the PLA film increased the films’ solubility up to 93%, while films containing CS oil extract maintained solubility values close to those of the control film. The increased film solubility is attributed to their higher extract content, a natural product that retains moisture, thus allowing more water to be absorbed upon immersion. Regarding the swelling degree, this is highly dependent on the type and extent of intermolecular chain interactions [56]. The swelling degree showed a reduction of ~30% for the film with the highest CS extract content (PLA-C3) and an increase of up to 63% for PLA-O6 film. It is possible that the interaction between CS extracts and the carboxyl groups of PLA reduced the availability of these groups to interact with water molecules, thereby influencing the swelling behavior. CS extracts, as particles in PLA matrix, also affect the physico-chemical properties of films. More specifically, films with unmodified coffee silverskin particles had similar values of solubility, in comparison to PLA films with CS chloroform extracts, and higher swelling degree values. Similar values for solubility and higher swelling degree have been observed for films with modified CS particles, in comparison to films with CS oil extract [39]. Coffee extracts, as well as cocoa and cinnamon, affect the physical properties of PLA films too, such as wettability and surface energy, probably caused by the introduction of polyphenols contained in these materials [5].

### 2.6. Mechanical Properties

**Figure 8:**
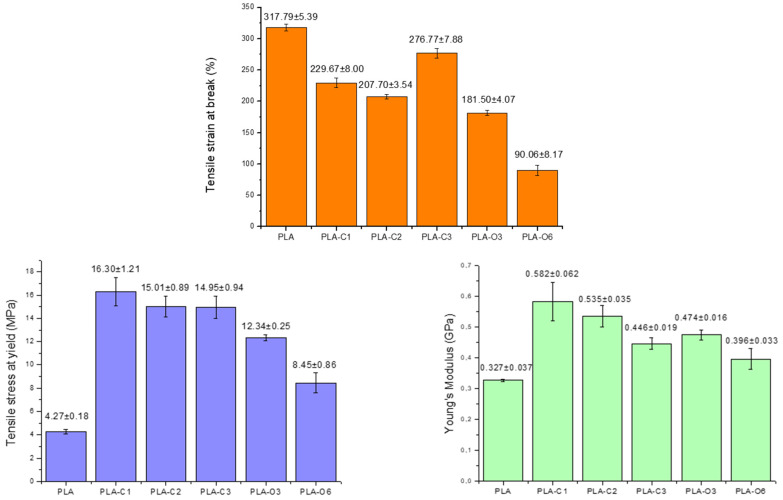
Mechanical properties of all composites prepared with coffee silverskin extracts.

Tensile testing was employed to examine the mechanical behavior of the films and the variations in tensile stress at yield, % tensile strain at break, and Young’s modulus shown in Figure 8. Comparing the samples containing CS chloroform extract, a significant decrease in tensile strain at break was observed, with PLA-C2 showing a reduction of ~35% compared to neat PLA. A notable decrease in tensile strain at break was also recorded in the case of CS oil-containing samples, with PLA-O6 showing the most significant reduction (90.6% compared to 317.8% for pure PLA). This reduction suggests that the CS oil extract restricts the ability of polymer chains to orient in the direction of applied stress, thereby limiting elongation at break. This conclusion aligns with DSC data, which evidenced enhanced crystallinity in films containing the CS extracts (Table 2).

All films showed a significant increase in tensile stress at yield with respect to pure PLA. The highest tensile stress at yield was recorded in the case of PLA-C1 film (16.3 ± 1.2 MPa, representing an increase of up to 288% over the PLA control. Similar values were reported for PLA films with spent coffee ground extracts [34]. PLA-O6 showed the lowest increase, with a tensile stress at yield of 8.45 ± 0.86 MPa, nearly double that of pure PLA (4.2 ± 0.17 MPa). As shown in Figure 8, the E-modulus of films containing both types of CS extracts was also higher than that of the PLA matrix.

Our films had higher values of tensile strain at break (%), tensile stress at yield (MPa), and Young’s modulus (GPa) in comparison with PLA films with pure CS particles in the matrix. Modified CS particles in PLA matrix led to higher levels of tensile strain at break (%) and tensile stress at yield (MPa), but similar Young’s modulus levels, with respect to films with CS extracts developed in this work [39]. PLA films loaded with green tea and rosemary polyphenolic extracts as an active packaging for almond and beef showed similar levels of tensile stress (MPa), higher levels of elongation (%), and lower levels of Young’s modulus compared to the films in this study [50]. PLA films containing an extract of Allium spp., intended for use in packaging, had a higher Young’s modulus and tensile stress and lower elongation, in contrast to films with CS extracts presented in this work [57]. Aqueous CS extracts in a wheat flour–glucose matrix led to reductions in tensile stress and Young’s modulus and did not affect the elongation of films [49].

Based on the findings, the developed mixtures could potentially be used in large-scale food packaging containers due to their improved mechanical properties, including ultimate tensile strength and Young’s modulus. However, it is important to note that these modifications may lead to a reduction in the elongation at break of PLA [58].

## 3. Materials and Methods

### 3.1. Materials

Coffee silverskin (CS), a byproduct of roasting Arabica coffee varieties, was supplied by AVEK S.A. (Thessaloniki, Greece), a local coffee roasting plant. Film-grade PLA Ingeo^TM^ Biopolymer 4043D was obtained from Nature Works LLC (Minnetonka, MN, USA), and was used as received. All other chemicals and solvents used were of reagent grade.

### 3.2. Extraction of Bioactive Compounds from Coffee Silverskin

**Figure 9:**
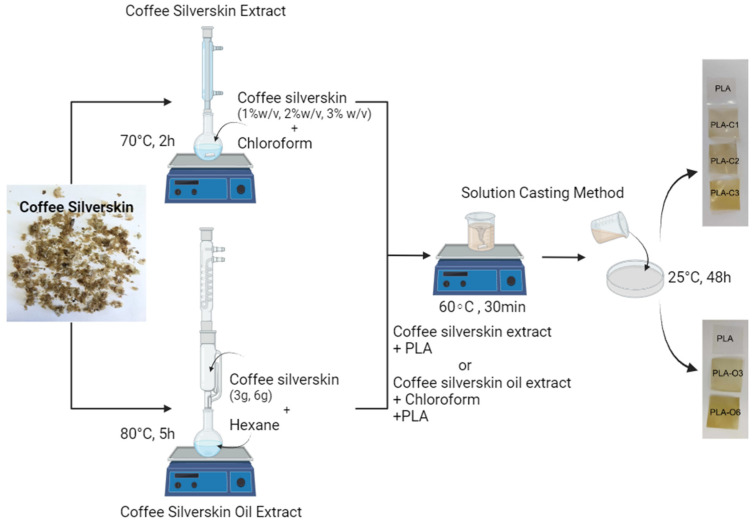
Preparation of PLA biocomposites with coffee silverskin extracts (created with BioRender.com).

Coffee silverskin (CS) powder, previously dried at 60 ± 2 °C for 24 h to remove moisture, was used to obtain either (i) a chloroform extract or (ii) oil via the Soxhlet extraction method. In the first case, three different solution extracts were prepared using 0.2, 0.4, and 0.6 g of CS powder in 20 mL of solvent (1, 2, and 3% *w*/*v*). CS was magnetically stirred in chloroform (CHCl_3_) at 70 °C for 2 h, using a flask equipped with a reflux column (Figure 9). The resulting suspension was vacuum-filtered, and the extracted solution was used as a solvent for solution casting with PLA pellets. For the Soxhlet extraction of coffee oil, 3 g and 6 g of dried CS were placed in a cellulose thimble. The extraction was carried out using 200 mL of n-Hexane as solvent, over a period of 6 h. After oil extraction, the hexane–oil mixture was transferred to a rotary evaporator to distill off the solvent. The oil fraction was then dried in an oven at 60 °C until constant weight. The oil yield was calculated using Equation (1), and the extraction was performed in triplicate.

(1) $$\%\;Yield=\frac{a}{b}\times 100$$

where *a* is the weight of the extracted oil (g) and *b* is the weight of dried CS (g).

### 3.3. Preparation of PLA/CS Biocomposite Films

PLA composite films were made using a solution casting technique. Using a magnetic hotplate stirrer, 1.5 g of PLA was added to the 20 mL chloroform solutions containing the coffee silverskin extracts (from the 1, 2, and 3% *w*/*v* initial suspensions) and heated to 60 °C for about 30 min while being continuously stirred. The resulting solution was then transferred onto flat glass plates. After that, the solvent was left to evaporate overnight at room temperature on a flat surface (films: PLA-C1, PLA-C2, PLA-C3). It should be noted here that the numbers 1, 2, and 3 in the symbol of the films qualitatively reflect the relative % of the extract in the polymer if it is assumed that the amount extracted is in the range of 8 to 10%, as measured in spent ground coffee samples. Further measurements (e.g., chromatographic via GC-MS or gravimetric via TGA) are needed in order to have accurate amounts of the compounds in the extract solutions. Two amounts of coffee silverskin oil from 3 g and 6 g dry coffee silverskin with 7.5% *w*/*v* chloroform solutions were heated to 60 °C for around 30 min under constant stirring on a magnetic hotplate stirrer. The solutions were poured on 11 cm diameter glass Petri plates. Chloroform removal was performed at room temperature overnight, before peeling the films from the mold (films: PLA-O3, PLA-O6). Below is a virtual presentation of the preparation of PLA biocomposite films (Figure 9).

### 3.4. Analytical Techniques

#### 3.4.1. Structural Analysis

Scanning Electron Microscopy (SEM) was used to examine the surface morphology of the films. The scanning electron microscope used in this work was the FESEM JSM 7610 FPlus (JEOL) (JEOL, Tokyo, Japan). The samples had to show conductivity in order to guarantee a correct analysis. The samples’ surface conductivity was increased by applying a tiny layer of carbon on them.

Fourier-Transform Infra-Red (FTIR) spectroscopy was employed to identify chemical interactions between PLA and CS. The FTIR measurements were performed using a Perkin Elmer Spectrum 1 spectrophotometer equipped with an attenuated total reflectance (ATR) device (instrument’s software Spectrum v5.0.1). Thin films were used for measurements, with spectra recorded from 4000 to 650 cm^−1^ at a resolution of 2 cm^−1^ (32 scans).

#### 3.4.2. Optical Properties

Color Measurements. A Macbeth CE 3000 spectrophotometer (Macbeth, London, UK) with a 10° standard observer with ultraviolet (UV) included and a specular component included was used to measure the colorimetric indicators under D65 illumination. Two folds were made to the samples. Positioned on a white calibration plate, the samples’ reflection spectra yielded their CIELAB coordinates. C* (Chroma) and hue angle (h) were also measured in our samples. Color strength of the films was examined using the Kubelka–Munk equation and a light reflectance approach, and the color strength (*K*/*S*) was determined as follows:

(2) $$\frac{K}{S}=\frac{{\left(1-R\right)}^{2}}{2R}$$

where *S* is the scattering coefficient, *K* is the absorption coefficient, and *R* is the reflectance at the highest absorption wavelength.

#### 3.4.3. Thermal Properties

Differential Scanning Calorimetry (DSC). The DSC-Diamond (Perkin-Elmer, Akron, OH, USA) was used to calculate the glass transition and melting point temperatures of each material produced. Approximately 5–6 mg of each sample was weighed, transferred to the usual Perkin-Elmer sample pan, sealed, and positioned on the instrument. Samples were heated to 200 °C at 10 °C per minute. The samples were cooled to 20 °C at a rate of 10 °C/min and then heated to 200 °C at a rate of 10 °C/min. The glass transition and melting point temperatures were then calculated.

#### 3.4.4. Functional Properties

Antioxidant activity was evaluated using the DPPH radical scavenging assay. A Shimadzu Spectrophotometer UV-1800 (Shimadzu, Kyoto, Japan) was used to perform the DPPH test in order to determine the antioxidant activity (*AA*) of the samples. Each 6 mg film sample was put in a vial with 3 mL of DPPH solution, and it was then left to incubate at 25 °C in the dark for a whole day. The following equation was used to calculate the films’ *AA*:

(3) $$AA\left(\%\right)=\frac{{ABS}_{control}-{ABS}_{sample}}{{ABS}_{control}}\times 100$$

where *ABS* denotes the absorption at 516 nm.

Oxygen Permeability. Using the N500 gas permeability analyzer model made by Guangzhou Biaoji Packaging Equipment Co., Ltd. (Guangzhou, China), the oxygen permeability of the produced films was evaluated. Specific parameters were followed during the evaluation, such as a steady temperature of 23 °C, 0% relative humidity, and a 10 mL/min gas flow rate.

Water vapor permeability test. Water vapor permeability was measured per ASTM E96 [59] using 6 cm diameter, 3 cm high glass Petri dishes filled with 10 mL of distilled water to create a 100% humidity environment. Films were secured to the dish edges with paraffin, leaving space above the water. The dishes were placed in a desiccator with silica gel and weighed initially and periodically for 24 h. Weight loss indicated the volume of water that permeated through the film, and permeability was calculated by plotting water passage over time and determining the slope (Δ*m*/Δ*t*). The assessment of the water vapor transmission rate (WVTR) was conducted utilizing the equation below, in which Δ*m*/Δ*t* represents the slope of the corresponding curve, and *A* denotes the area of the film.

WVTR = (Δ*m*/Δ*t*)/*A* (4)

The equation for water vapor permeability (WVP) is applicable when considering the film thickness represented by d_film_ and the pressure differential of the water vapor across the film, denoted as Δ*p* (T = 20 °C and Δ*p* = 2339 Pa).

*WVP* = (*WVTR* × d_film_)/Δ*p* (5)

Water content, film solubility and swelling degree.

A 1 cm by 1 cm section of the film was cut out, and its weight was recorded both before and after it was put in the oven (105 °C for 24 h) in order to determine the WC of the film with the following equation [60]:

(6) $$Water\;content\;\left(\%\right)=\frac{M_{o}-M}{M_{o}}\times 100$$

where *M_o_* is the initial mass (g) and *M* is the bone-dry mass (g).

The solubility and swelling properties of the films were evaluated using the methodologies described by Petaloti et al. [39]. To determine the initial dry mass (*M*_1_), film samples (1 cm × 1 cm) were dried in a vacuum oven for 24 h at 70 °C. The films were then sealed in plastic wrap, put in 50 mL beakers with 30 mL of distilled water, and kept at 25 °C for a whole day. Following the designated time, any water that was still in the beakers was disposed of, and filter paper was used to dry the residual film samples superficially. To calculate the final dry mass (*M*_3_), the leftover film samples (*M*_2_) were again dried in a vacuum oven for 24 h at 70 °C. For precision and uniformity, each film sample was measured three times.

(7) $$Film\;Solubility\;(\%)=\frac{M_{1}-M_{3}}{M_{1}}\times 100$$

(8) $$Swelling\;degree\;\left(\%\right)=\frac{M_{2}-M_{1}}{M_{1}}\times 100$$

#### 3.4.5. Mechanical Properties

A universal testing device, the Instron 3344 dynamometer, was used to measure tensile strength, elongation, and elastic modulus in compliance with ASTM D882 [61] at a crosshead speed of 50 mm/min. A Wallace cutting press was used to cut dumb-bell-shaped tensile test specimens, with the central parts measuring 5 × 0.5 mm in thickness and 22 mm in gauge length. The mean values of Young’s modulus, tensile strength at yield, and elongation at break were calculated by averaging the results of at least five measurements made for each sample.

### 3.5. Statistical Analysis

Every piece of data was examined three times. The properties of all the films under study were compared statistically using one-way analysis of variance (ANOVA). IBM SPSS Statistics 28 was used for statistical analysis. A *p*-value ≤ 0.05 was established as the threshold for statistical significance.

## 4. Conclusions

In this study, PLA-based films containing coffee silverskin extracts were successfully prepared using the solvent casting technique. The incorporation of coffee silverskin extracts into polylactide films enhances their water and oxygen barrier, mechanical strength, and antioxidant properties, making them suitable for advanced food packaging applications. Furthermore, the addition of CS extracts into the polymer matrix at any proportion did not affect the thermal properties (melting point and glass transition temperature) and the chemical structure of the polymer matrix. It can therefore be concluded that CS extracts had a positive effect on PLA properties, and they can be a potential alternative to synthetic additives in biodegradable packaging applications. This study demonstrates the potential of valorizing agro-industrial waste to develop sustainable and functional biocomposite materials, aligning with circular economy principles.

Further research could explore the scalability of the film preparation process and the biodegradability of the films under real-world conditions.

## Figures and Tables

**Table 1:** Color measurements of the PLA and biocomposite films studied.

	L*	a*	b*	c*	h	R% (400 nm)	K/S
PLA	91.58	−0.99	1.00	1.39	136.43	61.34	0.12
PLA-C1	82.98	−0.91	13.41	13.44	93.90	35.27	0.59
PLA-C2	78.63	0.73	16.64	16.66	87.47	25.03	1.12
PLA-C3	73.33	1.26	20.16	20.20	86.41	19.61	1.65
PLA-O3	64.64	2.79	27.54	27.68	84.21	11.85	3.28
PLA-O6	57.11	4.53	23.45	23.89	79.06	11.28	3.49

**Table 2:** Thermal characteristics of the biocomposite films studied.

	T_g_ (°C)	T_m_ (°C)	ΔH (J/g)
PLA	56.9	146.8	15.21
PLA-C1	58.1	146.7	18.07
PLA-C2	57.2	148.6	16.54
PLA-C3	57.3	148.3	17.33
PLA-O3	56.6	146.0/139.8	9.42
PLA-O6	57.1	147.4	13.10

**Table 3:** Oxygen transmission rate (OTR), water vapor transmission rate (WVTR), and water vapor permeability (WVP) of all studied composite materials.

	WVTR (g/m^2^·d)	WVP (10^−7^) (g/m·d·Pa)	OTR [cm^3^ mm/(m^2^·d·0.1 MPa)]
PLA	4.35	2.61	16
PLA-C1	3.61	2.01	9
PLA-C2	2.90	1.92	8
PLA-C3	2.61	1.40	7
PLA-O3	3.18	1.97	15
PLA-O6	2.58	1.49	13

**Table 4:** Physical properties of all composites prepared with coffee silverskin extracts. Different superscripts indicate statistically significant difference (*p* < 0.05).

	Water Content (%)	Solubility (%)	Swelling Degree (%)
PLA	8.11 ± 0.85 ^a^	0.71 ± 0.02 ^a^	1.19 ± 0.40 ^a^
PLA-C1	7.67 ± 0.39 ^a^	1.21 ± 0.30 ^b^	0.87 ± 0.33 ^b^
PLA-C2	8.68 ± 0.95 ^a^	1.27 ± 0.41 ^b^	0.85 ± 0.07 ^b^
PLA-C3	8.82 ± 0.25 ^a^	1.35 ± 0.43 ^b^	0.84 ± 0.23 ^b^
PLA-O3	7.93 ± 0.50 ^a^	0.69 ± 0.19 ^a^	1.56 ± 0.35 ^c^
PLA-O6	7.55 ± 0.62	0.72 ± 0.31 ^a^	1.94 ± 0.14 ^c^

## Data Availability

Data are available upon request from the corresponding author.

## References

[1] Matthews C., Moran F., Jaiswal A.K. (2021). A review on European Union’s strategy for plastics in a circular economy and its impact on food safety. J. Clean. Prod..

[2] Di J., Reck B.K., Miatto A., Graedel T.E. (2021). United States plastics: Large flows, short lifetimes, and negligible recycling. Resour. Conserv. Recycl..

[3] Ali S.S., Elsamahy T., Koutra E., Kornaros M., El-Sheekh M., Abdelkarim E.A., Zhu D., Sun J. (2021). Degradation of conventional plastic wastes in the environment: A review on current status of knowledge and future perspectives of disposal. Sci. Total Environ..

[4] Geyer R., Jambeck J.R., Law K.L. (2017). Production, use, and fate of all plastics ever made. Sci. Adv..

[5] Moraczewski K., Pawłowska A., Stepczyńska M., Malinowski R., Kaczor D., Budner B., Gocman K., Rytlewski P. (2020). Plant extracts as natural additives for environmentally friendly polylactide films. Food Packag. Shelf Life.

[6] Siracusa V., Rocculi P., Romani S., Rosa M.D. (2008). Biodegradable polymers for food packaging: A review. Trends Food Sci. Technol..

[7] Shogren R., Wood D., Orts W., Glenn G. (2019). Plant-based materials and transitioning to a circular economy. Sustain. Prod. Consum..

[8] Ramesh P., Prasad B.D., Narayana K.L. (2020). Effect of fiber hybridization and montmorillonite clay on properties of treated kenaf/aloe vera fiber reinforced PLA hybrid nanobiocomposite. Cellulose.

[9] Gironi F., Piemonte V., European Bioplastics (2011). Bioplastics Market Data 2018. Energy Sources Part A Recover. Util. Environ. Eff..

[10] Capanoglu E., Nemli E., Tomas-Barberan F. (2022). Novel Approaches in the Valorization of Agricultural Wastes and Their Applications. J. Agric. Food Chem..

[11] Murthy P.S., Naidu M.M. (2012). Sustainable management of coffee industry by-products and value addition—A review. Resour. Conserv. Recycl..

[12] Echeverria M.C., Nuti M. (2017). Valorisation of the Residues of Coffee Agro-industry: Perspectives and Limitations. Open Waste Manag. J..

[13] Hasballah K., Lestari W., Listiawan M.Y., Sofia S. (2022). Coffee by-products as the source of antioxidants: A systematic review. F1000Res.

[14] Bubonja-Sonje M., Giacometti J., Abram M. (2011). Antioxidant and antilisterial activity of olive oil, cocoa and rosemary extract polyphenols. Food Chem..

[15] Borrelli R.C., Esposito F., Napolitano A., Ritieni A., Fogliano V. (2004). Characterization of a New Potential Functional Ingredient: Coffee Silverskin. J. Agric. Food Chem..

[16] Ballesteros L.F., Teixeira J.A., Mussatto S.I. (2014). Chemical, Functional, and Structural Properties of Spent Coffee Grounds and Coffee Silverskin. Food Bioproc. Tech..

[17] Bessada S.M.F., Alves R.C., Oliveira M.B.P.P. (2018). Coffee silverskin: A review on potential cosmetic applications. Cosmetics.

[18] Bertolino M., Barbosa-Pereira L., Ghirardello D., Botta C., Rolle L., Guglielmetti A., Borotto Dalla Vecchia S., Zeppa G. (2019). Coffee silverskin as nutraceutical ingredient in yogurt: Its effect on functional properties and its bioaccessibility. J. Sci. Food Agric..

[19] Regazzoni L., Saligari F., Marinello C., Rossoni G., Aldini G., Carini M., Orioli M. (2016). Coffee silver skin as a source of polyphenols: High resolution mass spectrometric profiling of components and antioxidant activity. J. Funct. Foods.

[20] Hijosa-Valsero M., Garita-Cambronero J., Paniagua-García A.I., Díez-Antolínez R. (2018). Biobutanol production from coffee silverskin. Microb. Cell Fact..

[21] Toschi T.G., Cardenia V., Bonaga G., Mandrioli M., Rodriguez-Estrada M.T. (2014). Coffee silverskin: Characterization, possible uses, and safety aspects. J. Agric. Food Chem..

[22] Costa A.S.G., Alves R.C., Vinha A.F., Costa E., Costa C.S., Nunes M.A., Almeida A.A., Santos-Silva A., Oliveira M.B.P. (2018). Nutritional, chemical and antioxidant/pro-oxidant profiles of silverskin, a coffee roasting by-product. Food Chem..

[23] Mota D.A., Barbosa M.d.S., Schneider J.K., Lima Á.S., Pereira M.M., Krause L.C., Soares C.M.F. (2021). Potential Use of Crude Coffee Silverskin Oil in Integrated Bioprocess for Fatty Acids Production. JAOCS J. Am. Oil Chem. Soc..

[24] Efthymiopoulos I., Hellier P., Ladommatos N., Kay A., Mills-Lamptey B. (2019). Effect of Solvent Extraction Parameters on the Recovery of Oil From Spent Coffee Grounds for Biofuel Production. Waste Biomass Valoriz..

[25] Rocha M.V.P., de Matos L.J.B.L., de Lima L.P., Figueiredo P.M.d.S., Lucena I.L., Fernandes F.A.N., Gonçalves L.R.B. (2014). Ultrasound-assisted production of biodiesel and ethanol from spent coffee grounds. Bioresour. Technol..

[26] Kovalcik A., Obruca S., Marova I. (2018). Valorization of spent coffee grounds: A review. Inst. Chem. Eng..

[27] Singla P., Mehta R., Berek D., Upadhyay S.N. (2012). Microwave assisted synthesis of poly(lactic acid) and its characterization using size exclusion chromatography. J. Macromol. Sci. Part A Pure Appl. Chem..

[28] Wu C.S., Liao H.T. (2005). A new biodegradable blends prepared from polylactide and hyaluronic acid. Polymer.

[29] Kemsley E.K., Ruault S., Wilson R.H. (1995). Analytical, Nutritional and Clinical Methods Section Discrimination between Co&a arabica and Co&a canephora variant robusta beans using infrared spectroscopy. Food Chem..

[30] Lyman D.J., Benck R., Dell S., Merle S., Murray-Wijelath J. (2003). FTIR-ATR analysis of brewed coffee: Effect of roasting conditions. J. Agric. Food Chem..

[31] Paradkar M.M., Irudayaraj J. (2002). Rapid determination of caffeine content in soft drinks using FTIR-ATR spectroscopy. Food Chem..

[32] Craig A.P., Franca A.S., Oliveira L.S. (2012). Discrimination between defective and non-defective roasted coffees by diffuse reflectance infrared Fourier transform spectroscopy. LWT.

[33] Pujol D., Liu C., Gominho J., Olivella M., Fiol N., Villaescusa I., Pereira H. (2013). The chemical composition of exhausted coffee waste. Ind. Crop. Prod..

[34] Cacciotti I., Mori S., Cherubini V., Nanni F. (2018). Eco-sustainable systems based on poly(lactic acid), diatomite and coffee grounds extract for food packaging. Int. J. Biol. Macromol..

[35] Mota D.A., e Silva A.P.R., dos Santos J.C.B., Barbosa M.d.S., Lima Á.S., Krause L.C., Soares C.M.F. (2020). Extraction and Characterization of Coffee silverskin oil with potential application for enzymatic synthesis of fatty acids. Anais do VI Simpósio Internacional de Inovação e Tecnologia.

[36] Panpraneecharoen S., Chumanee S. (2020). Optimization of the oil extraction, study the chemical and physical properties of arabica spent coffee grounds. Sci. Technol. Asia.

[37] Raba D.N., Poiana M.A., Borozan A.B., Stef M., Radu F., Popa M.V. (2015). Investigation on crude and high-temperature heated coffee oil by ATR-FTIR spectroscopy along with antioxidant and antimicrobial properties. PLoS ONE.

[38] Chang Y.-C., Chen Y., Ning J., Hao C., Rock M., Amer M., Feng S., Falahati M., Wang L.-J., Chen R.K. (2019). No Such Thing as Trash: A 3D-Printable Polymer Composite Composed of Oil-Extracted Spent Coffee Grounds and Polylactic Acid with Enhanced Impact Toughness. ACS Sustain. Chem. Eng..

[39] Petaloti A.-I., Achilias D.S. (2024). The Development of Sustainable Biocomposite Materials Based on Poly(lactic acid) and Silverskin, a Coffee Industry By-Product, for Food Packaging Applications. Sustainability.

[40] Qin L., Qiu J., Liu M., Ding S., Shao L., Lü S., Zhang G., Zhao Y., Fu X. (2011). Mechanical and thermal properties of poly(lactic acid) composites with rice straw fiber modified by poly(butyl acrylate). Chem. Eng. J..

[41] Yasuniwa M., Tsubakihara S., Sugimoto Y., Nakafuku C. (2004). Thermal analysis of the double-melting behavior of poly(L-lactic acid). J. Polym. Sci. B Polym. Phys..

[42] Radjabian M., Kish M.H., Mohammadi N. (2010). Characterization of poly(lactic acid) multifilament yarns. I. The structure and thermal behavior. J. Appl. Polym. Sci..

[43] Giannakas A. (2020). Na-montmorillonite vs. organically modified montmorillonite as essential oil nanocarriers for melt-extruded low-density poly-ethylene nanocomposite active packaging films with a controllable and long-life antioxidant activity. Nanomaterials.

[44] Campos-Vega R., Loarca-Piña G., Vergara-Castañeda H.A., Oomah B.D. (2015). Spent Coffee Grounds: A Review on Current Research and Future Prospects.

[45] Bravo J., Juaniz I., Monente C., Caemmerer B., Kroh L.W., de Peña M.-P., Cid C. (2012). Evaluation of spent coffee obtained from the most common coffeemakers as a source of hydrophilic bioactive compounds. J. Agric. Food Chem..

[46] López-Galilea I., De Peña M.P., Cid C. (2007). Correlation of selected constituents with the total antioxidant capacity of coffee beverages: Influence of the brewing procedure. J. Agric. Food Chem..

[47] Mota D.A., Rajan D., Heinzl G.C., Osório N.M., Gominho J., Krause L.C., Soares C.M., Nampoothiri K.M., Sukumaran R.K., Ferreira-Dias S. (2020). Production of low-calorie structured lipids from spent coffee grounds or olive pomace crude oils catalyzed by immobilized lipase in magnetic nanoparticles. Bioresour. Technol..

[48] Ribeiro H.M., Allegro M., Marto J., Pedras B., Oliveira N.G., Paiva A., Barreiros S., Gonçalves L.M., Simões P. (2018). Converting Spent Coffee Grounds into Bioactive Extracts with Potential Skin Antiaging and Lightening Effects. ACS Sustain. Chem. Eng..

[49] Petaloti A.-I., Valtopoulou A., Gkogkou C., Achilias D.S. (2024). An Evaluation of the Use of Coffee Silverskin Particles and Extracts as Additives in Wheat Flour/Glucose Mixtures to Produce Bioactive Films for Food Packaging. Appl. Sci..

[50] Andrade M.A., Barbosa C.H., Cerqueira M.A., Azevedo A.G., Barros C., Machado A.V., Coelho A., Furtado R., Correia C.B., Saraiva M. (2023). PLA films loaded with green tea and rosemary polyphenolic extracts as an active packaging for almond and beef. Food Packag. Shelf Life.

[51] Fukushima K., Fina A., Geobaldo F., Venturello A., Camino G. (2012). Properties of poly(lactic acid) nanocomposites based on montmorillonite, sepiolite and zirconium phosphonate. Express Polym. Lett..

[52] Ali N.A., Noori F.T.M. (2014). Gas Barrier Properties of Biodegradable Polymer Nanocomposites Films. Chem. Mater. Res..

[53] Jang W.Y., Shin B.Y., Lee T.J., Narayan R. (2007). Thermal Properties and Morphology of Biodegradable PLA/Starch Compatibilized Blends. J. Ind. Eng. Chem..

[54] Ifezue C. (2009). The Effect of Bio-Based Materials on Quality and Shelf Life of Celery. Master’s Thesis.

[55] Peng Y., Wu Y., Li Y. (2013). Development of tea extracts and chitosan composite films for active packaging materials. Int. J. Biol. Macromol..

[56] Di Pierro P., Chico B., Villalonga R., Mariniello L., Damiao A.E., Masi P., Porta R. (2006). Chitosan-whey protein edible films produced in the absence or presence of transglutaminase: Analysis of their mechanical and barrier properties. Biomacromolecules.

[57] Llana-Ruiz-Cabello M., Pichardo S., Baños A., Núñez C., Bermúdez J., Guillamón E., Aucejo S., Cameán A. (2015). Characterisation and evaluation of PLA films containing an extract of Allium spp. to be used in the packaging of ready-to-eat salads under controlled atmospheres. LWT.

[58] Ljungberg N., Wesslén B. (2005). Preparation and properties of plasticized poly(lactic acid) films. Biomacromolecules.

[59] (2016). An Outline of Standard ASTM E96 for Cup Method Water Vapor Permeability Testing.

[60] Bakhshizadeh M., Ayaseh A., Hamishehkar H., Kafil H.S., Moghaddam T.N., Haghi P.B., Tavassoli M., Amjadi S., Lorenzo J.M. (2023). Designing a multifunctional packaging system based on gelatin/alove vera gel film containing of rosemary essential oil and common poppy anthocyanins. Res. Sq..

[61] (2005). Tensile Testing of Thin Plastic Sheeting.

